# Interleukin-15 enhances the proliferation, stimulatory phenotype, and antitumor effector functions of human gamma delta T cells

**DOI:** 10.1186/s13045-016-0329-3

**Published:** 2016-09-29

**Authors:** Heleen H. Van Acker, Sébastien Anguille, Yannick Willemen, Johan M. Van den Bergh, Zwi N. Berneman, Eva Lion, Evelien L. Smits, Viggo F. Van Tendeloo

**Affiliations:** 1 Laboratory of Experimental Hematology, Tumor Immunology Group (TIGR), Vaccine and Infectious Disease Institute (VAXINFECTIO), Faculty of Medicine and Health Sciences, University of Antwerp, Wilrijkstraat 10, 2650 Edegem, Antwerp Belgium; 2 Center for Cell Therapy and Regenerative Medicine, Antwerp University Hospital, Wilrijkstraat 10, 2650 Edegem, Belgium; 3 Center for Oncological Research (CORE), Faculty of Medicine and Health Sciences, University of Antwerp, Universiteitsplein 1, 2610 Wilrijk, Antwerp Belgium

**Keywords:** Adoptive transfer, Expansion protocol, Gamma delta T cells, Interleukin-15, Leukemia

## Abstract

**Background:**

Adoptive immunotherapy is gaining momentum to fight malignancies, whereby γδ T cells have received recent attention as an alternative cell source as to natural killer cells and αβ T cells. The advent of γδ T cells is largely due to their ability to recognize and target tumor cells using both innate characteristic and T cell receptor (TCR)-mediated mechanisms, their capacity to enhance the generation of antigen-specific T cell responses, and their potential to be used in an autologous or allogeneic setting.

**Methods:**

In this study, we explored the beneficial effect of the immunostimulatory cytokine interleukin (IL)-15 on purified γδ T cells and its use as a stimulatory signal in the ex vivo expansion of γδ T cells for adoptive transfer. The expansion protocol was validated both with immune cells of healthy individuals and acute myeloid leukemia patients.

**Results:**

We report that the addition of IL-15 to γδ T cell cultures results in a more activated phenotype, a higher proliferative capacity, a more pronounced T helper 1 polarization, and an increased cytotoxic capacity of γδ T cells. Moreover γδ T cell expansion starting with peripheral blood mononuclear cells from healthy individuals and acute myeloid leukemia patients is boosted in the presence of IL-15, whereby the antitumor properties of the γδ T cells are strengthened as well.

**Conclusions:**

Our results support the rationale to explore the use of IL-15 in clinical adoptive therapy protocols exploiting γδ T cells.

## Background

Cellular immunotherapy for cancer is a rapidly evolving field, endeavoring to deal with common relapse or resistance to conventional treatments. Herewith, most interesting are the γδ T cells, a T cell subset possessing a combination of innate and adaptive immune cell traits [1]. Activated γδ T cells have strong cytotoxic effector functions, by means of both death receptor/ligand and cytolytic granule pathways. Moreover, they have an immunoregulatory function, producing various cytokines, including the T helper (Th)1-associated cytokines tumor necrosis factor (TNF)-α and interferon (IFN)-γ [1]. γδ T cells are considered to be important players in cancer immune surveillance evidenced by; (a) the increased incidence of tumors in γδ T cell deficient mice [2, 3], (b) the overrepresentation of γδ T cells in reactive lymphatic regions associated with neoplasia, including acute myeloid leukemia (AML) [4], (c) their infiltration into solid tumors [5], and (d) their potential to kill a variety of tumor cells [1]. Pointedly, tumor-infiltrating γδ T cells recently emerged as the most significant favorable prognostic immune population across 39 malignancies [6]. As γδ T cells can kill tumor cells without previous contact and do not induce graft-versus-host disease, the adoptive transfer of γδ T cells could be a promising alternative for stem cell transplantation and adoptive transfer of αβ T cells [7].

Among the various tumor targets of γδ T cells [8], we focused on AML, a heterogeneous hematological malignancy involving the clonal expansion of myeloid blasts in the bone marrow and peripheral blood. Although significant improvement in treatment of AML has been made, the unfortunate reality is that currently available treatments are largely ineffective for most AML patients [9, 10]. In pursuit of new treatment options, several lines of evidence suggest that immunotherapy is an active modality in AML [11]. This includes the graft-versus-leukemia effect associated with allogeneic hematopoietic stem cell transplantation (HSCT), where it has been demonstrated that elevated γδ T cell immune recovery after HSCT is associated with a better outcome in terms of infections, graft-versus-host disease and overall survival [12–14]. Moreover, while the use of HSCT is restricted to a minority of patients due to, among other, its high transplant-related mortality and morbidity [10, 15], γδ T cell immune therapy is well-tolerated and safe [7, 16]. Adoptive transfer of γδ T cells is therefore an interesting alternative to tackle minimal residual disease and the high relapse rate in AML patients, optionally in combination with HSCT [17] or other (new) therapeutic agents [18–20]. Overall, while γδ T cell therapy holds great promise, clinical results are thus far modest, underscoring the need to further enhance the immunogenicity of the γδ T cell product [21].

γδ T cells can be expanded, using combinations of cytokines and phosphoantigens (e.g., isopentenyl pyrophosphate (IPP)) or aminobisphosphonates (e.g., zoledronate) [22]. The inclusion of aminobisphosphonates relies on their inhibition of farnesyl pyrophosphate synthase, a key enzyme of the mevalonate pathway, leading to accumulation of mevalonate metabolites such as IPP [23]. When peripheral blood mononuclear cells (PBMC) are treated with zoledronate, IPP will selectively accumulate in monocytes, due to the efficient drug uptake by these cells [24]. Therefore, in the absence of monocytes, addition of aminobisphosphonates is inefficient for induction of γδ T cell expansion, and the use of IPP or related phosphoantigen is required. To date, the standard protocol for the expansion of γδ T cells out of PBMC relies on the combination of zoledronate and interleukin (IL)-2 [25].

Limitations on the use of IL-2, relating to among other its toxicity, invigorate the investment in exploring other cytokines of the IL-2 family (also called the common γ chain cytokine family; IL-4, IL-7, IL-9, IL-15, and IL-21) [26]. IL-15 is of interest as it is closely related to IL-2 and has got the top position in the US National Cancer Institute’s ranking of 20 immunotherapeutic drugs with the greatest potential for broad usage in cancer therapy [27]. Moreover, both cytokines mediate their effects through a heterotrimeric receptor complex consisting of a cytokine specific α-chain (IL-2Rα or IL-15Rα), the IL-2/15Rβ-chain, and the common γ-chain [28]. IL-2 and IL-15 share many functions in regulating both adaptive (stimulation of T cell proliferation and induction of cytotoxic T lymphocytes) and innate (activating natural killer (NK) cells) immune responses [29]. In spite of the resemblances between both cytokines, they also have different functions in vivo. IL-15 is specifically known for its role in the maintenance of long-lasting, high-avidity T cell responses, whereas IL-2 may induce activation-induced cell death and provoke maintenance of regulatory T cells [26, 30]. Therewithal, IL-15 has shown efficacy in murine models of malignancy, even when IL-2 failed [31, 32]. Clinical trials with IL-15 as monotherapy have recently been initiated, investigating its therapeutic potential. When looking at the lymphocyte subsets in blood, both γδ T cell proliferation and activation were observed after IL-15 administration.

These results stressed the need for a detailed characterization of the sheer effect of IL-15 on untouched and isolated γδ T cells. The latter will be discussed in the first part of this paper, followed by the translation of the results into a γδ T cell expansion protocol for adoptive transfer.

## Methods

### Ethics statement and cell material

This study was approved by the Ethics Committee of the Antwerp University Hospital (UZA; Edegem, Belgium) under the reference number B300201419756. Experiments were performed using buffy coats derived from healthy volunteer whole blood donations (supplied by the blood bank of the Red Cross, Mechelen, Belgium) and blood samples from patients with AML obtained from the hematological division of the UZA (Table 1). PBMC were isolated by Ficoll density gradient centrifugation (Ficoll-Paque PLUS; GE Healthcare, Diegem, Belgium). Untouched γδ T cells were isolated from PBMC using the EasySep™ Human Gamma/Delta T Cell Isolation Kit (Grenoble, France), according to the manufacturer’s instructions. The purity of the γδ T cells was on average 94 % (min-max; 89–98 %). Isolated γδ T cells (1 × 10^6^ cells/mL) were cultured for 5 days in Iscove’s Modified Dulbecco’s Medium (IMDM, Life Technologies, Merelbeke, Belgium), supplemented with 10 % fetal bovine serum (FBS, Life Technologies), IPP (30 μg/mL; tebu-bio, Le-Perray-en-Yvelines, France), and IL-2 (100 IU/mL; Immunotools, Friesoythe, Germany) or IL-15 (12.5 ng/mL; Immunotools). The Burkitt’s lymphoma tumor cell line Daudi was kindly provided to us by the laboratory of Prof. Kris Thielemans (Free University of Brussels, Brussels, Belgium), and the multiple myeloma cell line U266 was a gift from Prof. Wilfred Germeraad (Maastricht University Medical Center, Maastricht, the Netherlands).

**Table 1:** AML patient characteristics

P	Sex	Age (years)	WHO type	Disease stage	PB blast %	BM blast %	Molecular	Cytogenetics
A	f	51	AML-nos	R1	6.5	16.2	WT1^+^	nl
B	m	51	AML-rga	Dx	50	64.4	NPM1^+^, WT1^+^	nl
C	m	62	AML-nos	Dx	2	72	ASXL1^+^	Trisomy 8
D	m	61	AML-nos	Dx CR1	47.8 0	79.8 0.6	Negative	Deletion 17p
E	f	76	AML-mds	Evolution from MDS	18	ND	ND	ND
F	m	52	AML-rga	Dx	92.5	94.5	WT1^+^, NPM1^+^, FLT3-ITD^+^	inv(3)(q21q26)
G	f	62	AML-nos	CR1	0	0.8	ASXL1^+^	trisomy 8

*AML* acute myeloid leukemia, *P* Patient, *f* female, *m* male, *WHO* World Health Organization (WHO) 2008 classification for AML, *AML-nos* AML not otherwise specified, *AML-rga* AML with recurrent genetic abnormalities, *AML-mds* AML with myelodysplasia (MDS)-related changes, *R1* first relapse of AML, *Dx* diagnosis stage, *CR1* first complete hematological remission of AML, *PB blast %* percentage of AML blasts in peripheral blood, *BM blast %* percentage of AML blasts in bone marrow, *ND* no data, *WT1* overexpression of Wilms’ tumor 1 (WT1) gene transcript, *NPM1* presence of mutated nucleophosmin 1 (NPM1), *ASXL1* presence of mutation in additional sex combs 1 (ASXL1) gene, *FLT3-ITD* presence of internal tandem duplication of fms-like tyrosine kinase 3 (FLT3), *nl* normal karyotype

### Proliferation assay

To test the ability of IL-2 and IL-15, in combination with IPP, to induce γδ T cell proliferation, a 5,6-carboxyfluorescein diacetate succinimidyl ester (CFSE; Invitrogen, Merelbeke, Belgium) flow cytometry-based proliferation assay was performed with isolated γδ T cells. Unstimulated CFSE-labeled γδ T cells served as negative control. After 5 days, cells were stained with LIVE/DEAD^®^ Fixable Aqua Stain (Life Technologies), CD56-PE (Becton Dickinson (BD); Erembodegem, Belgium), CD3-PerCP-Cy5.5 (BD), and γδ T cell receptor (TCR)-APC (Miltenyi) and analyzed using a FACSAria II cytometer (BD). γδ T cell proliferation was assessed by quantifying the percentage of proliferating (CFSE-diluted) cells within the viable (LIVE/DEAD^−^) CD3^+^γδTCR^+^ gate.

### Expansion protocol of γδ T cells (for adoptive transfer)

PBMC were resuspended in Roswell Park Memorial Institute (RPMI) supplemented with 10 % heat-inactivated human AB serum (Invitrogen, Merelbeke, Belgium), zoledronate (5 μM; Sigma-Aldrich, Diegem, Belgium), IL-2 (100 IU/mL), and/or IL-15 (100 IU/mL) at a final concentration of 1 × 10^6^ cells/mL. Cell cultures were maintained at a cell density of 0.5–2 × 10^6^ cells/mL and were replenished every 2 to 3 days by adding IL-2/IL-15-supplemented medium. Phenotypic and functional assays were performed on cells harvested at least 14 days after first stimulations.

### Immunophenotyping

Freshly isolated and 5-day proliferated γδ T cells were membrane-stained with the following monoclonal antibodies; γδ TCR-FITC (Miltenyi), CD56-PE (BD), CD69-PE (BD), and HLA-DR-PE (BD). Propidium iodide (PI; Life Technologies) was added to exclude dead cells from phenotypic analysis. Data acquisition was performed on a FACScan multiparametric flow cytometer (BD). Phenotypic characterization of γδ T cells was examined pre- and post-expansion, using CD27-FITC (BD), CD69-FITC (BD), CD56-PE (BD), CD80-PE (BD), CD45RA-PE-Cy7 (BD), CD28-PerCP-Cy5.5 (BD), CD16-PB (BD), CD86-V450 (BD), γδ TCR-APC (Miltenyi), and HLA-DR-APC-H7 (BD). Live/Dead^®^ Fixable Aqua Stain was used to distinguish viable from non-viable cells. Data were acquired on a FACSAria II flow cytometer (BD). Corresponding species- and isotype-matched antibodies were used as controls.

### Cytokine production

γδ T cell cultures were set up as described above. After 5 days of proliferation, cell-free supernatants were harvested and stored at −20 °C before analysis. Samples were assessed by using enzyme-linked immunosorbent assay (ELISA) for the presence of TGF-β (eBioscience, Vienna, Austria) and by using electrochemiluminescence immunoassay (ECLIA; Meso Scale Discovery (MSD), Rockville, MD, USA) for the presence of IFN-γ, TNF-α, IL-5, IL-10, and IL-17. Cytokine measurements were also performed on supernatant of γδ T cell cultures stimulated for an additional 4 h with the tumor cell lines Daudi and U266 at an effector-to-target (E:T) ratio of 5:1.

### Intracellular staining

After 14 days of γδ T cell expansion, IFN-γ and TNF-α production was measured using a flow cytometric-based intracellular staining assay. Measurements were also performed after an additional hour of stimulation with Daudi or U266 cells (E:T ratio = 5:1). Brefeldin A (Golgi-Plug 1 μL/mL; BD) was added to the different conditions (1 × 10^6^ cells/mL) and incubated for 3 h at 37 °C/5 % CO_2_. γδ T cells were then washed and incubated with Live/Dead^®^ Fixable Aqua Stain, CD3-PerCP-Cy5.5 (BD) and γδ TCR-APC (Miltenyi) for 30 min at 4 °C. Subsequently, cells were fixed and permeabilized, using the Foxp3/Transcription Factor Staining Buffer Set (eBioscience), according to the manufacturer’s instructions. Intracellular staining antibodies (IFN-γ-FITC and TNF-α-PE-Cy7, BD) or the corresponding isotype control were added and allowed to bind for 1 h at 4 °C.

### Cytotoxicity assay

A flow cytometry-based lysis assay was performed in order to determine the killing activity of γδ T cells against the tumor cell lines Daudi and U266. Tumor cells were labeled prior to co-culture with PKH67 Green Fluorescent Cell Linker dye (Sigma-Aldrich), according to the manufacturer’s protocol, and subsequently co-cultured with γδ T cells at different E:T ratios (1:10, 1:5, 1:1, 5:1, and 10:1). After 4 h, cells were acquired on a FACSAria II flow cytometer following staining with annexin V-APC (BD) and PI. Killing was calculated based on the percentages of viable (annexin V^−^/PI^−^) cells within the PKH67^+^ tumor cell population using the following equation: % killing = 100 − [(% viable tumor cells with γδ T cells/% viable tumor cells without γδ T cells) × 100].

### Statistics

Flow cytometry data were analyzed using FlowJo (v10; Treestar, Ashland, OR, USA). GraphPad Prism software (v5.0; San Diego, CA, USA) was used for statistical calculations and artwork. Shapiro-Wilk normality test was performed to ascertain the distribution of the data. *p* values <0.05 were considered statistically significant. All data are depicted as means ± standard error of the mean.

## Results

### Enhanced γδ T cell proliferation in response to IL-15, concomitant with an activated phenotype

**Fig. 1:**
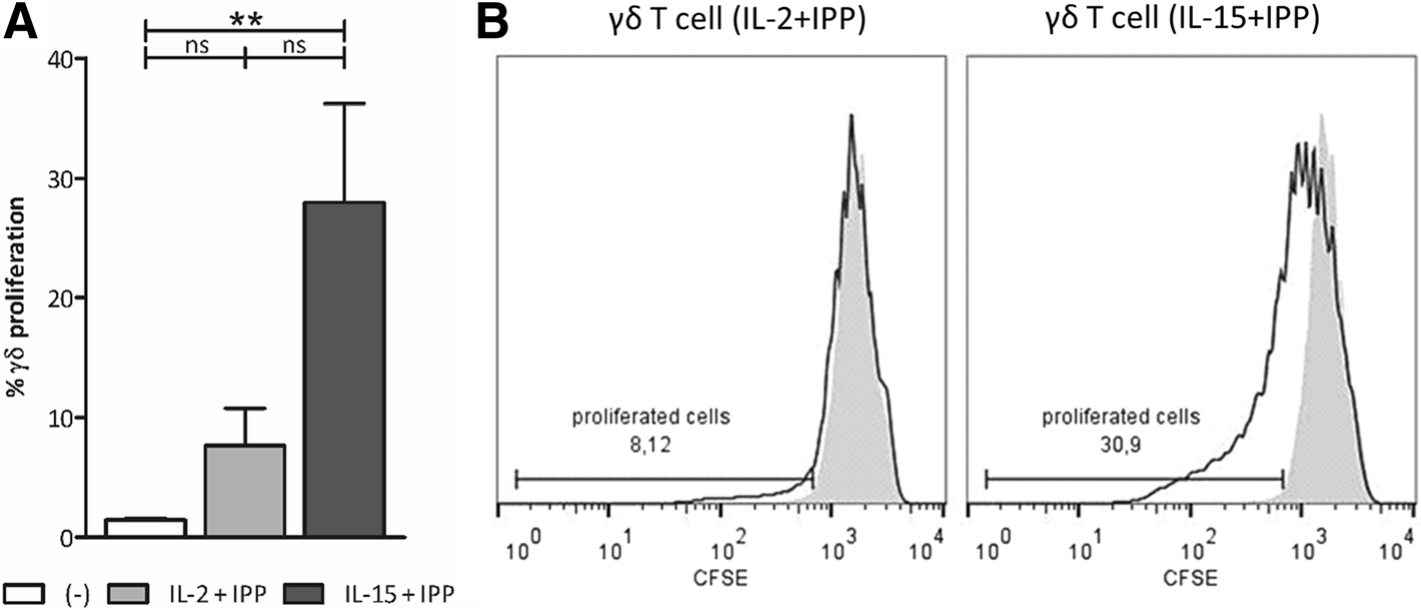
Purified γδ T cell stimulation with IL-15 and IPP for 5 days induces their proliferation. **a** Isolated γδ T cells were stimulated with IL-2+IPP (*gray bar*) or IL-15+IPP (*dark bar*) for 5 days. Unstimulated γδ T cells (*white bar*) were used as negative control. The percentage of proliferated (CFSE-diluted) cells within the viable γδ T cell population was determined by flow cytometry (*n* = 5) **b**. Histogram overlays show CFSE dilution and gating of unstimulated γδ T cells (*gray-filled area*) and γδ T cells exposed to IL-2+IPP or IL-15+IPP (*black line*) for one representative donor. Friedman test with Dunn’s multiple comparison test. **p < 0.01; *ns* not significant

Stimulation of αβ T cell proliferation is a characteristic of both IL-2 and IL-15. To assess their effect on γδ T cells, in combination with IPP, a 5-day proliferation assay was performed using isolated γδ T cells (Fig. 1). Unstimulated γδ T cells did not proliferate and were used as negative control. Although stimulatory effects were seen to some extent with the combination of IL-2+IPP, as evidenced by CFSE dilution, no statistical significance was reached relative to unstimulated γδ T cells. In contrast, IL-15+IPP stimulation induced distinct γδ T cell proliferation (Fig. 1). This finding was confirmed by a higher absolute number of γδ T cells after 5-day culture with IL-15+IPP. Calculating mean fold expansion, being the number of γδ T cells after stimulation divided by the number of γδ T cells at the start, IL-2+IPP and IL-15+IPP stimulation resulted in a mean fold expansion of 1.35 ± 0.15 x10^6^ and 2.18 ± 0.31 x10^6^ γδ T cells, respectively. Furthermore, exposure of human peripheral blood γδ T cells to IL-15+IPP resulted in an improved activated phenotype. Surface expression of CD69 augmented from 1.63 ± 0.52 % (basal level) to 15.85 ± 3.80 % (IL-2+IPP) and 26.55 ± 3.83 % (IL-15+IPP), and HLA-DR expression went up from 12.16 ± 2.36 % (basal level) to 64.58 ± 5.63 % (IL-2+IPP) and 63.93 ± 7.63 % (IL-15+IPP). Notably, CD56 expression was significantly higher on IL-15+IPP stimulated γδ T cells (38.34 ± 4.83 %) than on unstimulated (19.20 ± 4.49 %) and on IL-2+IPP stimulated γδ T cells (28.14 ± 4.73 %).

### IL-15+IPP stimulated γδ T cells display strong Th1 cytokine responses and are more effective killers than IL-2+IPP γδ T cells

**Fig. 2:**
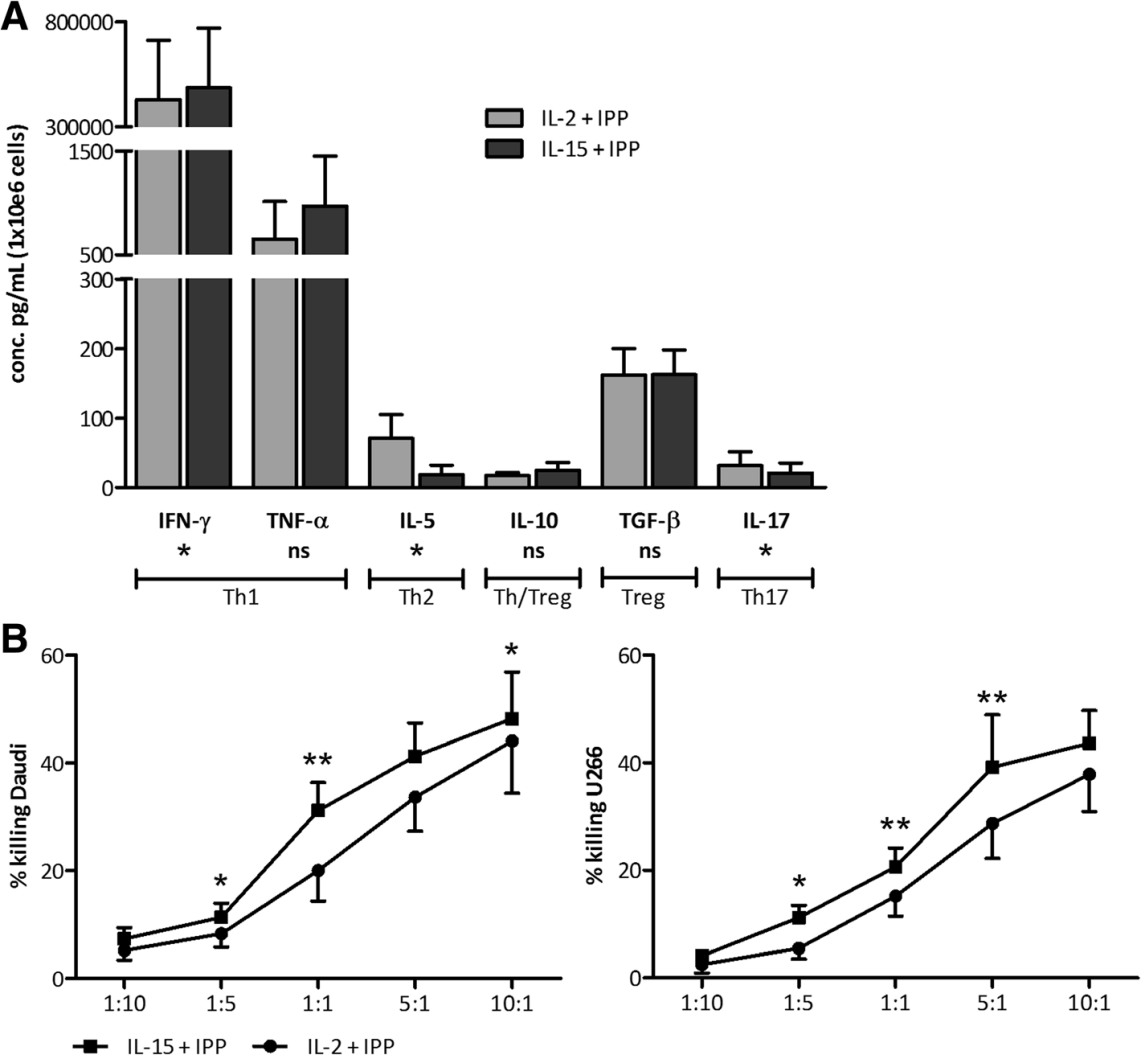
Distinct Th1 cytokine release and marked tumor cell killing by IL-15+IPP activated γδ T cells. **a** γδ T cell invigorated for 5 days with IL-15 and IPP (*dark bar*) or IL-2 and IPP (*gray bar*) were stimulated for 4 h with U266 cells (E:T = 5:1). *Bar graphs* represent the cytokine secretion level of 1 million γδ T cells (pg/mL), as determined by ECLIA (IFN-γ, TNF-α, IL-5, IL-10, and IL-17) and ELISA (TGF-β). Data are depicted as mean of duplicate measurements of six independent donors. Wilcoxon matched-pairs signed rank test. **b** γδ T cells stimulated for 5 days with IL-15+IPP (*filled squares*) or IL-2+IPP (*filled circles*) were analyzed by flow cytometry for cytotoxicity against the tumor cell lines Daudi and U266. Target cell killing was determined by annexin V/PI staining after 4-h incubation at different E:T ratios. Results are expressed as mean percentage killing, calculated using the formula specified in the “Methods” section. Data are from six different experiments involving 6–12 different donors. Paired *t* test. **p < 0.01; *p < 0.05

An important question in the context of cancer immunotherapy is whether IL-15, as compared to IL-2, can boost γδ T cell effector functions, in terms of cytokine secretion and cytotoxic capacity. γδ T cells stimulated with either IL-2+IPP or IL-15+IPP are characterized by a largely pro-inflammatory cytokine profile comprising IFN-γ and TNF-α. IFN-γ levels were typically >68 ng/mL/1 × 10^6^ γδ T cells. On average, 3512 ± 2140 and 4506 ± 2527 pg/mL/1 × 10^6^ γδ T cells of TNF-α was detected in supernatant of 5-day cultures of IL-2+IPP and IL-15+IPP activated γδ T cells, respectively. With the exception of the IL-5 concentration, for which a 4.4-fold higher concentration was produced by IL-2+IPP γδ T cells, no significant difference was found in the non Th1 cytokine secretion (IL-10, IL-17 and TGF-β tested) between both γδ T cell types. After 5 days, γδ T cell cultures were harvested and exposed to Daudi or U266 tumor cells, to verify their antitumor activity, including cytokine secretion (Fig. 2a) and cytolytic capacity (Fig. 2b). The same trends were observed as prior to tumor cell exposure, i.e., a clear Th1 response for both IL-2 and IL-15-cultured γδ T cells. However, IL-15+IPP γδ T cells gave rise to significantly higher concentrations of IFN-γ compared to IL-2+IPP γδ T cells, whereas the latter were more prone to a Th2 and Th17 response. As direct antitumor activity is a hallmark of γδ T cells, and an important asset for immunotherapy, γδ T cell-mediated cytotoxicity was tested next against Daudi and U266 cells. γδ T cell cytotoxicity augmented with increasing E:T ratios and γδ T cells from all donors were able to kill both tumor cell lines. In addition, γδ T cell-mediated lysis was unambiguously stronger for the IL-15+IPP stimulated γδ T cells in comparison with the IL-2+IPP stimulated ones against both Daudi and U266 (Fig. 2b).

### IL-15 boosts IL-2/zoledronate-mediated expansion of γδ T cells

**Fig. 3:**
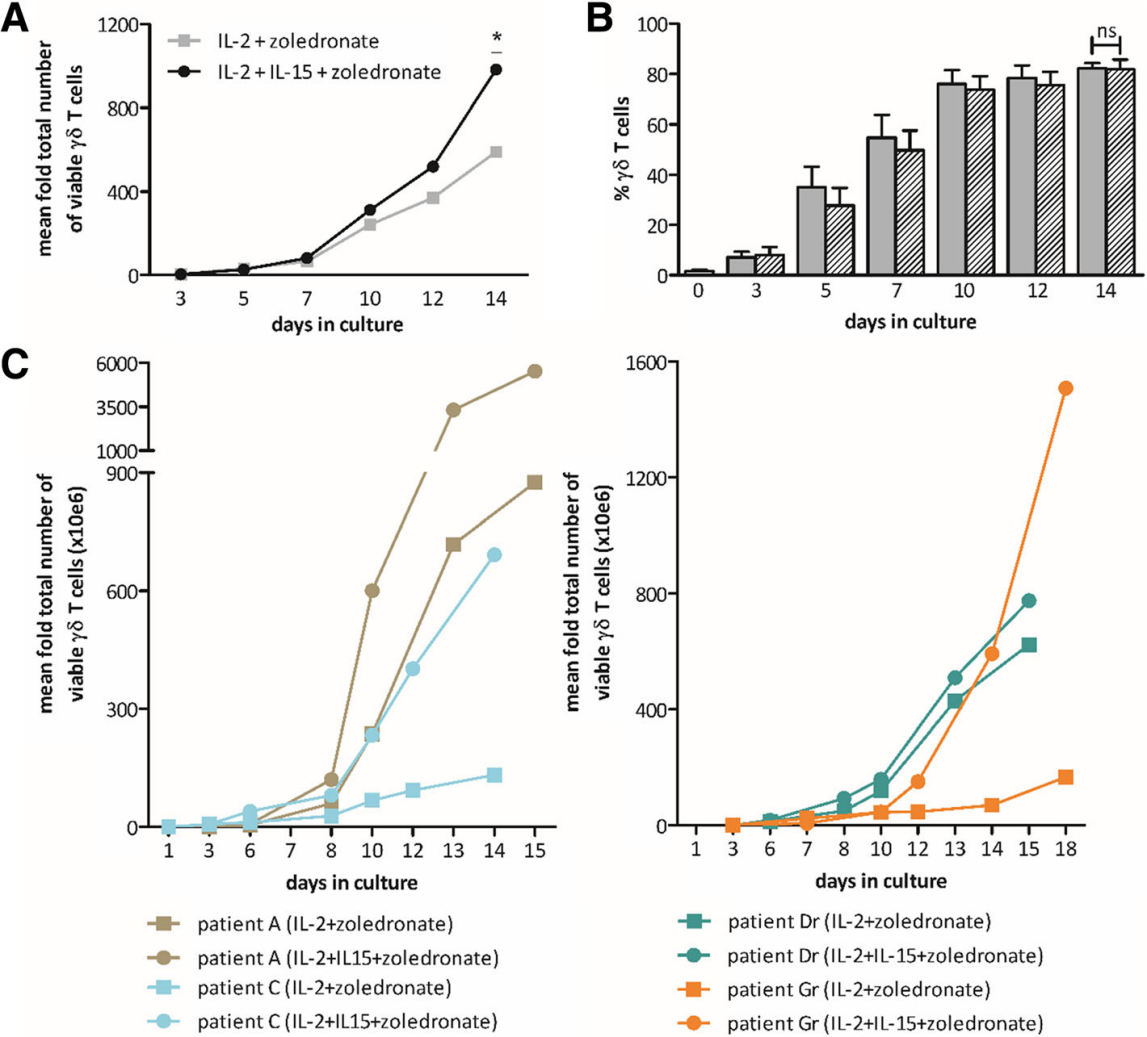
Kinetics of γδ T cell expansion. **a** The graph represents the average (*n* = 6, healthy donors) mean fold total number of γδ T cells, calculated as the absolute number of γδ T cells at the respective time point divided by the absolute number of γδ T cells at start of the expansion culture. **b** Bar graphs showing the proportion of γδ T cells at various times during the culture, starting from day 0. Cultures expanded with zoledronate and IL-2 are displayed as *gray bars*, and cultures supplemented with zoledronate, IL-2, and IL-15 are shown as *hatched bars*. Data are expressed as mean percentages of 6 healthy donors from three experiments. **c** The increase in number of “successfully” cultured γδ T cells from different AML patients is presented as mean fold total number of viable γδ T cells. Patients in remission are indicated by a lowercase r after their letter of identification (right panel). Wilcoxon matched-pairs signed rank test. *P* patient; *p < 0.05; *ns* not significant

As a first step in the clinical translation of the preceding results towards an improved protocol for the expansion of γδ T cells for adoptive transfer, expansion based on stimulation of PBMC with IL-2 and zoledronate was compared with responsiveness to IL-15 and zoledronate. Of the four healthy donors tested, only half of the cultures stimulated with IL-15 and zoledronate succeeded. One culture was considered to have failed due to a culture viability of less than 50 % and another one due to the absence of γδ T cell expansion. Yet, IL-2+zoledronate-mediated γδ T cell expansion occurred in all four donors. On the other hand, when IL-15 was added as a third stimulus to the standard expansion protocol (IL-2+zoledronate), a clear effect was detected (Fig. 3). Despite the fact that the percentage (Fig. 3b) and viability of γδ T cells after 14 days were similar between cultures stimulated with IL-2+zoledronate or IL-2+IL-1 zoledronate, a significant increase in total γδ T cell yield was observed when IL-15 was added to the cultures (Fig. 3a). On average, IL-2+zoledronate cultures showed a 590-fold increase in γδ T cells after 14 days, whereas addition of IL-15 almost led to a 1000-fold increase. To ascertain the strength of the immune proliferative and stimulatory effect of IL-15, immune cells of AML patients (Table 1) were included. First, AML patients had a significantly lower proportion of γδ T cells in PBMC in comparison with healthy donors (0.30 ± 0.13 versus 1.91 ± 0.37 %, respectively; *p* = 0.0234). Next, it should be noted that in contrast to the 100 % success rate of γδ T cell expansion (defined as ≥70 % γδ T cells after culture [33, 34]) starting from PBMC of healthy donors, less than half of the γδ T cell expansion cultures from AML patients succeeded (Table 2). However, no direct correlation could be made between γδ T cell percentages in PBMC at start and the success of the expansion culture. Cultures that expanded relatively poorly, but in which nonetheless sufficient (viable) γδ T cells were induced for (functional) testing, were also included. Importantly, similar to the healthy donors, γδ T cells expansion was convincingly stronger for all AML patients when IL-15 was added to the cultures (Fig. 3c).

**Table 2:** Outcome of in vitro expansion of γδ T cells from PBMC of AML patients

P	% γδ T cells IL-2+zoledronate	% γδ T cells IL-2+IL-15+zoledronate	Success
A	75.9	91.9	Yes
B	16.8	12.4	No
C	84.4	94.0	Yes
D	17.2	46.6	No
Dr	76.6	89.4	Yes
E	5.65	1.80	No
F	4.31	1.84	No
Gr	27.1	50.8	No

*P* patient, *Pr* patient in remission

### Fourteen-day expansion using IL-2+IL-15+zoledronate gives rise to γδ T cells with an activated memory phenotype

**Fig. 4:**
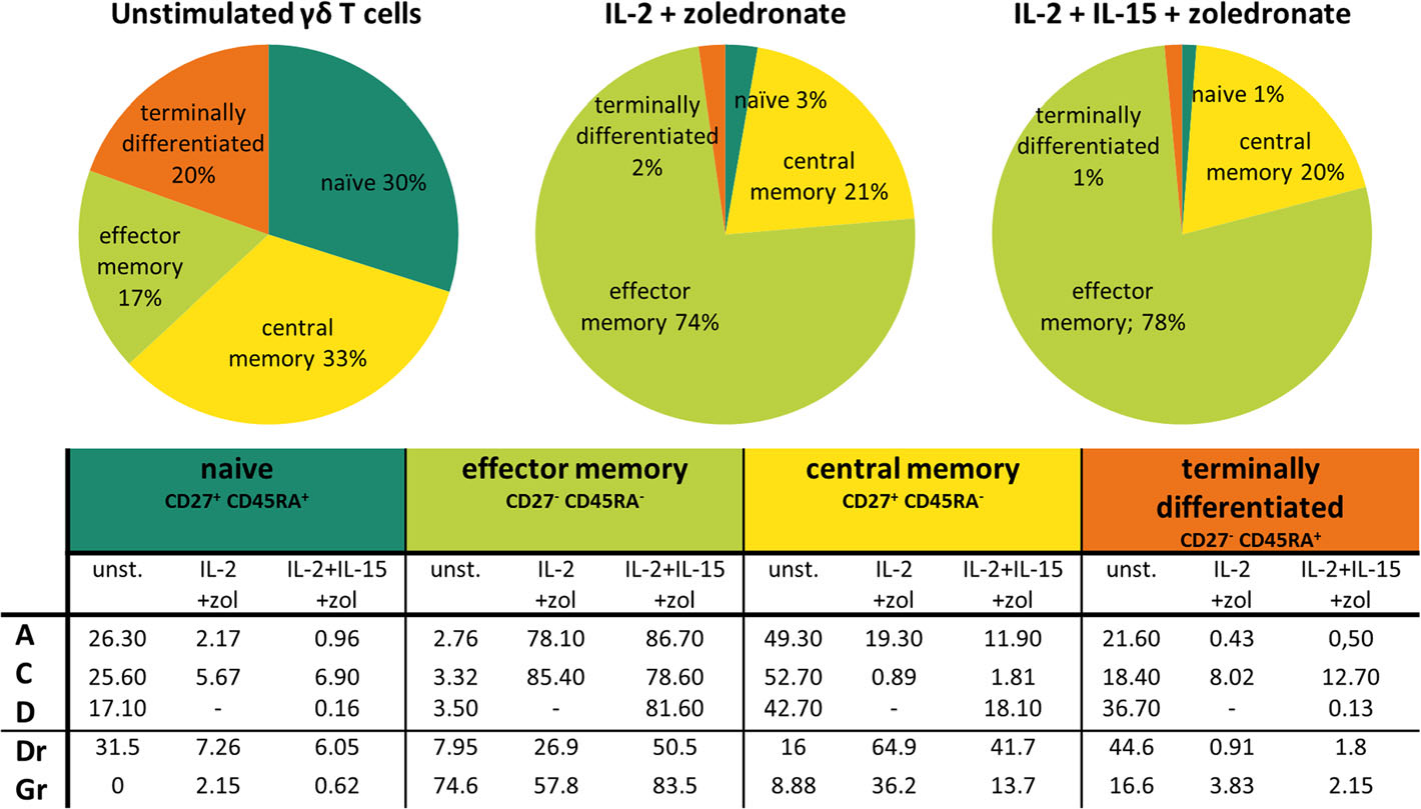
Effector memory phenotype of γδ T cells pre- and post-expansion. Average percentage of naïve (CD45RA^+^CD27^+^), effector memory (CD45RA^−^CD27^−^), central memory (CD45RA^−^CD27^+^) and terminally differentiated (CD45RA^+^CD27^−^) γδ T cells, before (unstimulated) and after expansion, represented as pie charts for six healthy donors. Values of γδ T cells of patients are listed in the table below. - no value due to low cell number, *Pr* patient in remission, *unst.* unstimulated, *zol* zoledronate

**Fig. 5:**
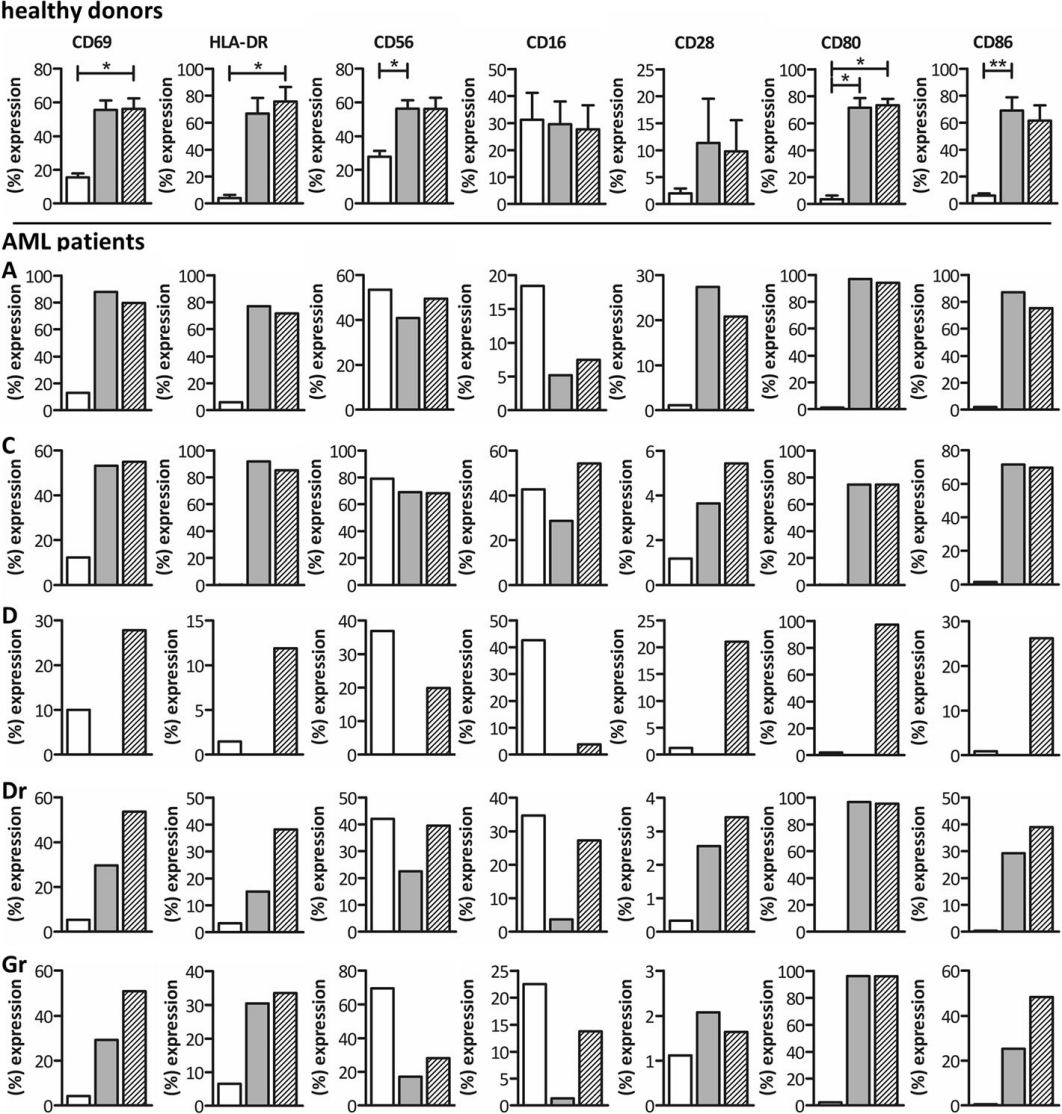
γδ T cell phenotype prior and subsequent to expansion. Comparison of the expression of CD69, HLA-DR, CD56, CD16, CD28, CD80, and CD86 on unstimulated (*white bars*), IL-2+zoledronate expanded (*gray bars*) and IL2+IL-15+zoledronate expanded (hatched bars) γδ T cells. Results are shown for six healthy donors on the uppermost row as mean percentage. Expression levels of the respective markers of patient γδ T cells are showed below. Due to the low number of γδ T cells after expansion with IL-2 + zoledronaat of patient D, no data are available. Friedman test with Dunn’s multiple comparison test. **p < 0.01; *p < 0.05

At culture initiation (day 0) and termination (day 14–15), we subjected the γδ T cell cultures to immunophenotyping. Whereas the four distinct effector/memory states were nearly evenly represented in terms of percentages by circulating γδ T cells of healthy donors at day 0, with a small preponderance for the naïve and central memory phenotype, the vast majority of expanded cells were effector memory γδ T cells and to a lesser extent central memory γδ T cells (Fig. 4). γδ T cells of AML patients, however, deviated from this pattern (Fig. 4). Likely, the stage of the disease, i.e., diagnosis, relapse or remission, plays a role in this phenomenon. For example, notwithstanding the rather small patient group, patients at diagnosis or in relapse displayed at day 0 a higher amount of central memory γδ T cells as compared to healthy donors, whereas remission patients strikingly had less central memory γδ T cells. Undoubtedly, patient-dependent factors also play a role, for instance when looking at the distribution of patient G (Fig. 4). Still, in analogy with healthy donors, expanded γδ T cells of AML patients show a clear predominance for the memory stages (Fig. 4). Further phenotypic characterization revealed a marked increase in expression level of the activation associated markers CD56, CD69 and HLA-DR on expanded γδ T cells of healthy donors, in combination with an increase in the co-stimulatory molecules CD80 and CD86 (Fig. 5). Expansion, however, had no effect on the expression of CD16 and CD28. If we then looked at the phenotype of patient-derived γδ T cells after expansion, we perceive an increase in the early and late activation markers CD69 and HLA-DR as well (Fig. 5). Interestingly, γδ T cells of patients in remission express less HLA-DR (and CD69) after expansion in comparison to healthy controls and patients with acute disease. The activated phenotype of γδ T cells from remission patients is nonetheless clearly improved upon addition of IL-15. With regard to CD56 expression, all patient γδ T cells exhibited already elevated levels at start, similar to those of healthy donors after expansion. After expansion, a down-regulation of CD56 is seen, which is more pronounced in patients in remission and γδ T cells expanded with IL-2+zoledronate. The same phenomenon holds true for CD16 upon expansion. Concerning the expression of CD80, a marked upregulation was detected after expansion, even more pronounced as compared to healthy donors. Whereas the expression of CD86 is similar for patient A, patient C, and healthy donors, γδ T cells of patients in remission expressed less of this co-stimulator after expansion (Fig. 5). In summary, expanded γδ T cells display an activated memory phenotype, based on the enhanced expression of CD69, HLA-DR, CD56, CD80, and CD86.

### The frequency of IFN-γ-producing γδ T cells is considerably higher after IL-2+IL-15+zoledronate expansion compared to IL-15-free expansion

**Fig. 6:**
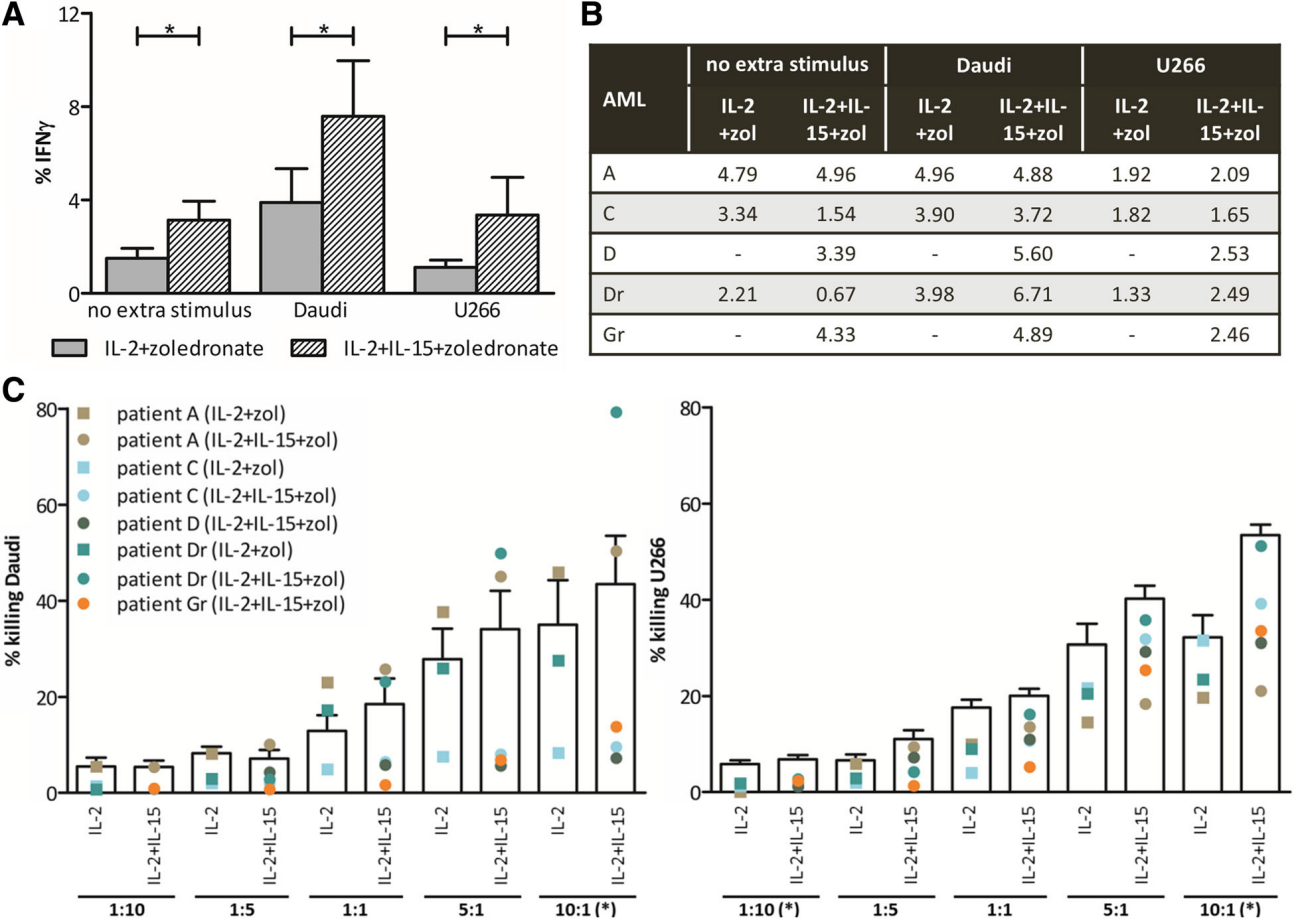
Pro-inflammatory and cytolytic γδ T cell capacity after expansion. Percentage IFN-γ-positive γδ T cells after expansion (no extra stimulus) and after an additional 4-h stimulation with Daudi or U266 cells (E:T ratio = 5:1) measured by flow cytometry for **a** six healthy donors and **b** five AML patients. **c** Expanded γδ T cells, with IL2+zoledronate (IL-2+zol) or IL-2+IL-15+zoledronate (IL-2+IL-15+zol), were analyzed by flow cytometry for cytotoxicity against Daudi and U266. Target cell killing was determined by annexin V/PI staining after 4-h incubation at different E:T ratios. *Bar graphs* (*white*) represent the mean percentage killing of six healthy donors in three independent experiments. The *dots* display the proportion of tumor cell killing by γδ T cells of the different patients. Wilcoxon matched-pairs signed rank test. - no value due to low cell number; *p < 0.05

To study the functionality of the expanded γδ T cells, we first determined their Th1 polarization. In the absence of additional stimuli, the mean frequency of IFN-γ-producing γδ T cells after expansion was significantly higher when stimulated with IL-2+IL-15+zoledronate (3.28 ± 0.81 %), as compared to IL-2+zoledronate (1.49 ± 0.43 %) (Fig. 6a). This more pronounced Th1 profile of γδ T cells, seen when IL-15 is present during expansion, also holds true upon addition of tumor cells to the cultures. When Daudi cells were added, we even observed an amplified IFN-γ production as compared to the control (no tumor cells) condition (Fig. 6a). Despite having low numbers of TNF-α-producing γδ T cells, generally below 1 %, the same trends were seen as with IFN-γ. Proceeding with immune cells of AML patients, different observations were seen as compared to expanded γδ T cells of healthy donors (Fig. 6b). Whereas the fraction of γδ T cells positive for IFN-γ after stimulation with IL-2+IL-15+zoledronate was comparable between healthy donors and AML patients, IL-2+zoledronate stimulation gave rise to more IFN-γ positive γδ T cells when derived from AML patients relative to healthy donors. Moreover, the IL-2+zoledronate cultures of AML patients performed equally well in terms of IFN-γ production, if not better, than the IL-2+IL-15+zoledronate condition. Next, when tumor cells were added to expanded γδ T cells from patients, blunting of the γδ T cell helper function could be observed to a certain degree pertaining to healthy donor expanded γδ T cells. Here, γδ T cells of patients with active disease did similar in terms of percentage IFN-γ positive cells after expansion with or without IL-15. On the other hand, γδ T cells of remission patient Dr, of which both protocols had yielded sufficient γδ T cells for functional testing, exhibited an almost doubling in the number of IFN-γ positive cells when expanded in the presence of IL-15 and challenged with tumor cells.

### Addition of IL-15 to the expansion medium results in a significant increase in the γδ T cell-mediated cytotoxicity against tumor cells

Finally, we determined the cytotoxic potential of the expanded γδ T cells against the Daudi and U266 tumor cell lines (Fig. 6c). γδ T cells of healthy donors displayed an increase in tumor cell killing upon increasing E:T ratios. Notably, addition of IL-15 in the expansion cultures evoked a superior cytotoxic capacity of expanded γδ T cells at the 10:1 ratio for both tumor cell lines. Similarly, the cytotoxic capacity of γδ T cells originating from AML patients was improved upon expansion in IL-15. However, in general, killing values of AML-derived γδ T cells were lower than those of healthy donor γδ T cells. At the 10:1 ratio, average killing of Daudi by expanded γδ T cells was 27.26 ± 10.86 and 32.03 ± 14.17 %, with respectively the IL-2+zoledronate (*n* = 3) and IL-2+15+zoledronate (*n* = 5) expansion protocols. For U266 killing, percentages of 24.89 ± 3.51 and 35.19 ± 4.96 % were true. Finally, for some AML patients e.g., patient A, cytotoxic capacity is highly dependent on the target cell line (Fig. 6c).

## Discussion

To date, adoptive immunotherapy using T lymphocytes has been applied for nearly 30 years [35]. Since then, vast improvements, including the use of chimeric antigen receptor (CAR) or T cell receptor (TCR) gene-modified killer T cells, have been made. This resulting in proven clinical benefit for end-stage cancer patients for which standard therapy failed and the provision of long-term protection in some cases [36–38]. Notwithstanding this progress in the field, one of the major problems encountered with the adoptive transfer of classical αβ T cells, gene-modified or not, is the occurrence of off- and on-target toxicity [37]. Here, γδ T cells have received recent attention as an alternative cell source for T cell-mediated anticancer therapy [7]. Namely, both adoptive transfer and in vivo activation/expansion of γδ T cells are safe therapeutic modalities that can result in objective clinical responses in the treatment of cancer [7, 16]. A major advantage with using γδ T cells is that they are unlikely to cause graft-versus-host disease, allowing them to be generated from healthy donors and given in an allogeneic setting as an “off-the-shelf” therapeutic [7, 14]. In this study, we explored the beneficial effect of IL-15 on γδ T cells and its use as stimulatory signal in the ex vivo expansion of γδ T cells for adoptive transfer. The strength of IL-15-mediated activation was further validated in γδ T cells originating from AML patients.

A recent study in rhesus macaques showed that continuous administration of IL-15 is, among other, associated with increased numbers of circulating γδ T cells [39]. This has now been confirmed in the first-in-human trial of recombinant IL-15, whereby IL-15 administration in cancer patients induced both γδ T cell proliferation and activation [26]. These in vivo findings corroborate with our in vitro results, showing substantial proliferation of isolated γδ T cells upon stimulation with IL-15+IPP, but not with IL-2+IPP. In line herewith, Viey et al. demonstrated γδ T cell expansion out of tumor-infiltrating lymphocytes of renal cell carcinoma patients with bromohydrin pyrophosphate, a synthetic phosphoantigen, in combination with IL-15, while the combination with IL-2 was inefficient [5]. However, when we sought to extrapolate these results into a clinical 2-week expansion protocol based on IL-15 and zoledronate, inconsistencies in expansion successes were detected. In our hands, the best results were obtained when IL-15 and IL-2 were combined, suggesting that these cytokines have distinct roles in γδ T cell biology. An advantage of IL-15 during expansion could be that IL-15 mediates homeostatic competition between αβ T cells, NK cells and γδ T cells by a yet unknown indirect mechanism [40, 41]. While we detect no difference in the final percentage of γδ T cells in expansion cultures of healthy donors with or without IL-15, this is certainly the case with the cultures of AML patients. In the IL-2+zoledronate cultures, we observed a higher proportion of other blood mononuclear cells, of which the vast majority were αβ T cells. Addition of IL-15 may therefore have counteracted the growth of other cell types such as αβ T cells, constraining the growth of the γδ T cells, preventing cluster formation and subsequently successful γδ T cell expansion [40, 41]. It is possible that this process is more important when there is only a low percentage of γδ T cells in the starting material or when γδ T cells are less responsive, which is often the case in a malignant setting [42]. Moreover, γδ T cells of AML patients generally exhibited a higher viability after expansion with IL-15, which was not observed with healthy donors. The question remains whether this improved viability is a result or the cause of the enhanced degree of γδ T cell expansion. Nevertheless, it has been shown that IL-15 is very competent in supporting survival of activated γδ T cells, superior to IL-2 [43], and T cell survival in general [44]. This does not mean that IL-2 is by definition redundant, as evidenced by our expansion results. The synergism between IL-2 and IL-15 could lie in the fact that γδ T cells, in response to zoledronate and IL-2, upregulate their expression of *inter alia* IL-2Rβ and γ_c_ [43].

With regard to the effector/memory state of the γδ T cells after expansion, our results correspond with the available literature concerning γδ T cells expanded with IL-2 and zoledronate [25, 45]. In particular, the majority of expanded γδ T cells from healthy donors and patients showed for both expansion protocols a predominant effector memory type and, to a lesser extent, a central memory phenotype. Hence, the addition of IL-15 had no apparent influence on the effector/memory state. This is encouraging, since increased effector memory γδ T cells are reported to correlate with objective clinical outcomes in patients treated with zoledronate and IL-2 [46]. On the other hand, adoptive transfer with αβ T cells has shown that it may also be important to preserve some less differentiated T cell subsets within the infused T cell product, to ensure T cell expansion and potentially long-term T cell persistence [47–49]. Given the presence of γδ T cells with a central memory phenotype upon expansion, our culture protocol fulfills the above-mentioned necessity. Moreover, contributing to the rationale of implementing IL-15 in expansion protocols, it has recently been shown that IL-15 instructs the generation of human memory stem T cells, a T cell subset with superior antitumor responses [50], from naive precursors [49, 51].

When looking in detail at the phenotype of cultured γδ T cells, two findings stand out from our results. First, incubation with IL-15+IPP led to a significant upregulation of CD56 relative to IL-2+IPP stimulated and unstimulated γδ T cells. In addition to the stringent association of CD56 with NK cells, CD56 has also been detected on other lymphoid cells, including γδ T cells and activated CD8^+^ T cells [52–55]. Moreover, CD56 in the human hematopoietic system is not restricted to lymphoid cells. Both CD56^+^ plasmacytoid dendritic cells (DCs) and myeloid DCs, including our IL-15 DCs, feature cytotoxic activity, in addition to serving classical DC functions [56]. In this context, it has been presumed that CD56 is associated with activated/cytotoxic effector immune cells [52, 54, 57–59]. This would implicate that IL-15 stimulation raises stronger cytotoxic γδ T cells, as effectively confirmed by the enhanced killing of tumor cells in our experiments. Moreover, CD56 expression on γδ T cells following expansion was enhanced as well. Secondly, upon expansion, an increased expression of the co-stimulatory molecules CD80 and CD86 was observed. It has been shown that activated γδ T cells are able to acquire a professional antigen-presenting cell function, expressing high levels of co-stimulatory molecules [60]. This function may further boost the generation of a potent and long-lasting immune response.

In view of the functionality of γδ T cells, our experiments clearly show that IL-15, in comparison with IL-2, has a superior Th1 polarizing effect on γδ T cells and markedly strengthens the γδ T cell cytotoxic capacity. These results are supported by in vivo data where intestinal γδ T cells of IL-15^−/−^ knockout mice only produced small amounts of IFN-γ upon stimulation and showed significantly lower cytotoxicity against target cells as compared to wild-type mice [61]. This indicates that IL-15-mediated signals are indeed indispensable for the development of potent antitumor functions. Furthermore, IL-15 has proved itself superior at maintaining effector functions of already expanded and activated γδ T cells, evidenced by a higher IFN-γ production and CD107a expression after stimulation with zoledronate pre-treated Daudi cells [43]. In addition, within the context of neonatal immunity, it has been shown that expansion of cord blood γδ T cells with alendronate and IL-15 gave rise to γδ T cells capable of strong protective immune responses [62]. Although the differences between the effects of IL-2 and IL-15 were subtle, higher expression of among other granzyme B and perforin was detected after activation with IL-15 [62]. All these data therefore point towards the fact that IL-15 is a pivotal signal to generate more powerful effector γδ T cells. This has recently been substantiated by Ribot et al., identifying the MAPK/ERK-mediated IL-2/IL-15 signaling as the major functional differentiation pathway of human γδ T cells towards antitumor (cytotoxic type 1) effector cells [63].

## Conclusions

Taken together, we have demonstrated that the addition of immunostimulatory cytokine IL-15 to in vitro γδ T cell cultures, derived from both AML patients and healthy blood donors, resulted in a more activated phenotype and significantly increased the antitumor functions of γδ T cells. This was manifested in a higher yield of expanded γδ T cells, a more pronounced Th1 polarization and an increased cytotoxic capacity, all desirable characteristics for their further use in adoptive immunotherapy. Therefore, our results strengthen the rationale to explore the use of IL-15 in clinical adoptive therapy protocols.

## Abbreviations

AML: Acute myeloid leukemia
CFSE: 5,6-carboxyfluorescein diacetate succinimidyl ester
DC: Dendritic cell
E:T: Effector-to-target
FBS: Fetal bovine serum
HSCT: Hematopoietic stem cell transplantation
IFN: Interferon
IL: Interleukin
IMDM: Iscove’s Modified Dulbecco’s Medium
IPP: Isopentenyl pyrophosphate
NK: Natural killer
PBMC: Peripheral blood mononuclear cells
PI: Propidium iodide
RPMI: Roswell Park Memorial Institute
TCR: T cell receptor
Th: T helper
TNF: Tumor necrosis factor
